# Supplementary material for: Airway epithelial SARS-CoV-2 infectious and repair responses: relationships to age, sex, and post-COVID pulmonary syndromes
Source: bioRxiv. 2025 Sep 2:2025.07.17.663733. Originally published 2025 Jul 17. Preprint. [Version 2] doi: 10.1101/2025.07.17.663733 (PMC12338744; doi:10.1101/2025.07.17.663733)
Supplement: Supplement 1 [file media-1.pdf]

Supplementary Table 1. Features of cell cultures used in this study.

Donor No.	Donor ID	Batch**	Age (yr)$	Sex	Ancestry
1	Y-58P	1	4 mo	M	Caucasian
2*	Y-15K	1	10 mo	M	Caucasian
#3	Y-37O	1	2	M	Caucasian
4	Y-48G	1	5	F	Caucasian
5	Y-49O	1	8	F	Hispanic
#6	M-9N	1	21	M	African American
7	M-10H	1	26	M	Caucasian
#8	M-25O	1	26	F	Caucasian
9	M-19O	1	27	M	Unknown
#10	M-74K	1	29	M	Caucasian
11	E-5I	1	69	M	African American
12	E-10G	1	70	F	Caucasian
#13	E-35K	1	70	M	Caucasian
14	E-26P	1	73	F	Caucasian
#15	E-3N	1	91	M	Caucasian
16	Y-18I	2	2 mo	F	Caucasian
17	Y-46M	2	4 mo	F	Caucasian
18	Y-83K	2	10 mo	M	African American
19	M-35P	2	21	F	Caucasian
20	M-51K	2	21	F	Hispanic
21	M-21M	2	23	F	Caucasian
22	E-41T	2	69	F	Unknown
23	E-30T	2	70	M	Unknown
24	E-11G	2	78	F	Caucasian

*This sample was excluded from the Protocol 1 dataset due to experimental/technical errors during viral infection.
**This batch number refers to Protocol 1 only; Protocol 2 was conducted in one batch.
NOTE: in addition to the above footnote, the following two samples were excluded from the RNAsequencing analyses due to failure to pass QC testing: Donor #1, 1 dpi mock; and Donor #11, 14 dpi mock.
#For the study of metabolites, these donors were used.
$mo=months for donors < 1 year old.
