# Supplementary material for: Airway Epithelial SARS-CoV-2 Infectious and Repair Responses: Relationships to Age, Sex, and Post-COVID Pulmonary Syndromes
Source: bioRxiv. 2025 Jul 17:2025.07.17.663733. Preprint. [Version 1] doi: 10.1101/2025.07.17.663733 (PMC12338744; doi:10.1101/2025.07.17.663733)
Supplement: Supplementary file 1 [file media-1.pdf]

Supplemental Material:

Methods and Figures for Dang, H. et al. Airway Epithelial SARS-CoV-2 Infectious and Repair Responses: Relationships to Age, Sex, and Post-COVID Pulmonary Syndromes

Methods Section. This section contains detailed experimental and technical details related to the assays and data analyses that are presented in the main text.

A. Experimental Design for *In Vitro* Studies

The experimental design is outlined in **Supplemental Figure 1**. In the first *in vitro* studies (referred to in main text as Protocol 1), air–liquid interface (ALI), well-differentiated HBE cultures (≥ 4 wks after plating) were infected with SARS-CoV-2 (D614G) at an MOI of 0.5 at 0 dpi and followed for 14 days, with samples/cultures collected for analyses as indicated (1). Three age groups (N = 8/group) were studied: Young (age 0-8); Mature (age 21-29): Elderly (> 69). The first experiment was conducted in two batches, which are outlined in **Supplemental Table 1**. The combined but not individual (first batch age- but not sex-matched; second batch compensated for first batch sex-mismatched) produced a study that had equal representation across all three age groups for both sexes. A second replication protocol studied the same donors in a single batch over a 3 dpi interval (**Supplemental Figure 1B**). Details of the donor specifics are provided in **Supplemental Table 1.**

B. Infection Protocol & Titering

The ALI-cultured HBE cells were exposed to SARS-CoV-2 recombinant viruses (D614G strain) as described previously (2). In brief, 200ul of a SARS-CoV-2 virus solution was applied onto apical culture surfaces at 37°C for 2 hours at an MOI of 0.5. Following incubation, the virus solution was removed, cells washed twice with 500 µl of PBS. The infected cells were then incubated at 37°C for 1, 3, 7, or 14 days post infection (dpi). For "mock" cultures, incubation with apical 200 µl PBS was performed. The basolateral cell culture media was changed every 48 hpi (hours post infection) and collected at 1, 3, 7, 10, and 14 dpi. For viral titer measurements, 200 µl PBS was applied onto the apical surface of cultures and allowed to incubate for 10 min at 37°C, followed by apical aspiration. Titering was performed in duplicate on apical washes collected at 2 hpi, and 1, 2, 3, 7, and 14 dpi utilizing virus plaque assays. Apical PBS washings were serially diluted 10^{-1} to 10^{-6} in PBS and 200 µL of each diluted sample applied to wells of a 6-well plate containing Vero-E6 cell monolayers. Plates were incubated at 37°C for 1 hour. Following incubation, each well was overlaid with a 1:1 mixture of agarose and 2X DMEM. Plates were then left to solidify and incubated at 37°C for 3 days. Plaques were visualized with neutral red stain at day 3 and counted as plaque-forming units per ml (PFU/ml). Details of the live virus titers for Protocol 1 are provided in **Supplemental File 1**. Details of live viral titers for Protocol 2 are found in **Supplemental File 2.**

C. Identification of viral genetic variation via viral RNA-sequencing

Purified RNA was prepared from cell lysates for sequencing using the Illumina Stranded Total RNA Prep (Illumina, 20040525) workflow. The resulting libraries were run on a MiSeq instrument with at least 1 million reads per sample. Reads were aligned to the SARS-CoV-2 genome using STAR (v2.7.9). Output BAM files were input into Geneious Prime, and variant

calling was performed with a minimum of 100 reads per base, a quality score of 25, and a cutoff of 0.02 frequency variance. Variants were graphed along the length of the genome using a custom R script. Details of the mutations found and the mutation load are found in **Supplemental File 3**).

D. Morphology

Immunostaining of HBE cell cultures was performed as described previously (3, 4). Sections were baked at 60°C for 2 hours, followed by deparaffinization with xylene for 10 minutes x 3, and rehydration with graded ethanol exposures (100%, 95% and 70% ethanol for 5 minutes each). Quenching of endogenous peroxidase was performed with 0.5% hydrogen peroxide in methanol for 15 minutes. Antigen retrieval was performed by microwaving slides in 0.1 M sodium citrate pH 6.0 (3 cycles with 1200W microwave settings: 100% power for 6.5 minutes, followed by 60% power for 6 minutes twice). The slides were then washed with PBS and blocked with Blocking One buffer (03953-95, Nacalai Tesque) at room temperature for 30 minutes. The primary antibodies utilized were a rabbit anti-MUC5B (H-300, Santa Cruz, 1:1000 dilution) and mouse anti-MUC5AC (45M1, Invitrogen, 1:1000 dilution) applied to the slides and incubated at 4℃ overnight. The sections were washed with PBS-T (phosphate-buffered saline containing 0.1% Triton X-100) and incubated with secondary antibodies (biotinylated donkey anti-rabbit IgG [711-065-152, Jackson ImmunoResearch, 1:400 dilution or goat anti-mouse IgG [115-065-166, Jackson ImmunoResearch, 1:400 dilution]). The slides were then treated with Vectastain Kit (PK-4000, Vector Laboratories) followed by visualization with DAB and counterstaining with Fast Red. The images were acquired as described above. Isotype controls were prepared using rabbit IgG (011-000-003, Jackson ImmunoResearch) and/or mouse IgG (015-000-003, Jackson ImmunoResearch).

Stained images were scanned and digitized using an Olympus VS200 microscope with a 20X 0.80 N.A., 40X 0.95 N.A., or 60X 1.42 N.A. objectives. Stained and total cell area was determined by Fiji software for quantification.

E. Immunohistochemistry Combined with TUNEL Staining

Immunohistochemistry (IHC) and TUNEL staining were performed sequentially on mock and infected HBE sections from 6 donors at 3- and 14-dpi. Briefly, IHC and TUNEL were performed using SARS-CoV-2 (Abclonal A18797), Click-iT^{TM} Plus TUNEL Assay (Invitrogen, Carlsbad, California, Cat #C10618), and DAPI for nuclear stain. Fluorescence was detected with a Leica Stellaris5 Confocal microscope. Total cell, SARS-infected, and apoptotic cells were counted per field of view (FoV) and normalized to mock infected cultures. Individual TUNEL results are found in **Supplemental File 4**.

F. Apical Surface Proteomics of HBE Cultures

Apical surface washes of infected cultures were collected using 250 microliters of PBS per culture, inactivated using 8M urea, and prepared for label-free quantitative proteomic analysis using the filter-aided sample preparation (FASP) (5). The protocol included the reduction of cysteine residues by 10 mM dithiothreitol (Sigma-Aldrich) and alkylation in 50 mM iodoacetamide (Sigma-Aldrich). Proteins were digested using trypsin (25 ng/µl) overnight at 37°C. The resulting peptide mixtures were freeze-dried and dissolved in 30 µl of 2% acetonitrile and 0.1% trifluoroacetic acid. For each sample 1 µl was injected for chromatography tandem mass spectrometry (LC-MS/MS) analysis, utilizing a Q-Exactive (Thermo Scientific) mass spectrometer coupled to an Ultimate

3000 nano HPLC system (Thermo Scientific). Data acquisitions were performed as described in Kesimer et al. 2015 (6).

The collected MS/MS raw data were processed and searched against the UniProt human protein database (December 2021) using Proteome Discoverer 1.4 software (Thermo Scientific) and against the UniProt viral data base SARS CoV2 2019 (April 2022) using the Mascot (Matrix Science) search algorithm (7). [The following parameters were used in the Sequest and Mascot search engines: 10 ppm mass accuracy for parent ions and 0.02 Da accuracy for fragment ions; 2 missed cleavages were allowed. Carbamidomethyl for cysteines was set as a fixed modification, and methionine oxidation was set to variable. Scaffold 4.7.5 (Proteome Software Inc.) was used to validate MS/MS-based peptide and protein identifications. Peptide identifications were accepted if they had greater than 95.0% probability by the scaffold local FDR algorithm. Protein identifications were accepted if they had greater than 99.0% probability and contained at least 2 identified peptides. Protein probabilities were assigned by the Protein Prophet algorithm (8). Proteins that contained similar peptides and could not be differentiated based on MS/MS analysis alone were grouped to satisfy the principles of parsimony. The relative protein quantities are expressed as the total precursor intensities of all their assigned peptides.

G. Proteomic Data Processing

Raw data as label-free quantities (LFQ) of normalized total precursor intensity from different timepoints were compiled based on unique gene names for the proteins. The LFQ matrix was log2 transformed, which converted zeros as missing values. Proteins/genes were filtered with >30 non-missing values (out of total 5 timepoints x 23 codes = 115 samples) for further analysis. The

log2LFQ values were normalized with median centering to 0, and the missing values were imputed by left-censored quantile regression or QRILC using the Bioconductor R packages, *MsCoreUtils* (9), and *imputeLCMD* (https://cran.r-project.org/web/packages/imputeLCMD). The normalized and imputed data set was used for subsequent analyses. Details of the proteomics data are provided in **Supplemental File 5**.

H. Bulk RNAseq and data analysis

Each insert was left in TRI reagent (T9424, Sigma-Aldrich) for >24 hours to deactivate viral infectivity. Total RNA was extracted according to the manufacturer's instruction (R2052, Zymo Research). Library preparation (polyA selection) and sequencing were conducted by Genewiz (South Plainfield, NJ). Paired-end sequencing reads in FASTQ format were mapped to the human reference genome, GRCh38, supplemented with the SARS-CoV-2 genome (NC_045512) and GENCODE release 37 human and ENSEMBL v101 SARS-CoV-2 gene annotations, using the STAR aligner (10). Gene expression estimates from the alignment BAM files were made using the transcriptome assembler, StringTie v2.1.5 (11), and outputs were compiled as Transcripts Per Million (TPM) mapped reads and raw counts. Positive strand viral RNAs were quantified based on Open Reading Frame (ORF) boundaries. Since all canonical viral transcripts end at the 3'-end of the viral genome, TPM of a viral RNA gene/ORF represents specific transcript plus the overlapping 3'-UTRs of all upstream viral genes. Accordingly, TPMs for ORF1ab represent canonical genomic RNA. The total of the last known expressed canonical sub-genomic reads for nuclear capsid protein (designated N) approximates the total viral TPM. Differential expression (DE) analyses were performed using the dream function from the Bioconductor R package, *variancePartition* (12) and linear mixed-effect models with donor code

as the random-effect factor. Differential gene expression data are provided separately for each analyses in Supplemental Files (see **Supplemental Table 2** for details). The following covariates were included in all bulk-seq DE models: Batch for batch-effect, PC1 (Principle Component #1) for data quality, median gene expression of 348 ciliated marker genes derived from airway single-cell data set (13) for cell differentiation status. Principle Component Analyses (PCA) were performed using the R package *ade4* (14). Hierarchical clustering heatmaps were generated using the Bioconductor R package C*omplexHeatmap* (15), and various plots were made with the R package *ggplot2* (16). Gene expression data has been deposited in GEO (Accession #: GSE299837). Sample level calculations of viral TPM are found in **Supplemental File 1**. Genes associated with each heatmap shown in the manuscript are listed in **Supplemental File 6**.

I. scRNAseq data analysis: GSEA methods:

Single-cell RNA-seq data analysis from airway brushing samples were described as before (17). Briefly, epithelial cells were subset from the final data set and exported as mtx data matrix and meta-data, which were imported into R. Differential gene expression between pulmonary long COVID vs controls was performed using the R package, *NEBULA* (18), accounting for gender and age as covariates.

I. GSEA methods:

Gene set enrichment analysis (GSEA) was performed using log2 fold-change ranked genes from differential gene expression results from bulk RNA-seq, scRNA-seq, and proteomic analyses. Protein-coding genes that survived minimal expression filter for bulk RNA-seq, or scRNA-seq and proteomic analysis were ranked by logFC for comparisons of interests. The pre-ranked

GSEA was then performed using the Bioconductor R package, *fgsea* (19), with gene set collection obtained from Reactome pathways (20). Custom pathways and gene lists of interests were curated manually from published literature. The custom pathways were appended to Reactome pathways for analyses. Custom pathway details are provided in **Supplemental File 7.** The analyses results for all Reactome pathways + the custom pathways are shown for each relevant comparison (between male and female, between young and mature, between young and old for mock and virus across dpi) in Supplemental Files (see **Supplemental Table 2** for details).

J. Metabolite levels

Levels of adenosine triphosphate (ATP) metabolites and methionine salvage pathway metabolites were determined using a previously described LC-MS/MS method (21, 22). Sample level data for metabolite values are shown in **Supplemental File 8.**

I. Analyses of 14 dpi repair/failed repair

A composite pathway index was generated from the 12 pathways utilized to describe the disease repair at 14 dpi. Genes from 12 selected pathways related to tissue repair were used to generate the "repairIndex" following 2 steps: a) averaging TPM values from genes per pathway to generate 1 pathway index per sample; b) combine the pathway index using a formula (Type1ISGs + NuclEncodedMito + HistoneAcetylLE + KEGGcytokReceptor + TP53cellCycleLE + ReactEGFRsigLE + SenescencePPI + CFTRtransportors + MucusSecretion – HBEciliated + HBEdeuterosome + antiOxidant) of addition of pathway indices, except for "HBEciliated" genes with subtraction since ciliated cells were diminished by viral infection, and complete repair is expected to restore them to parity with mock treated samples. This index was

tested against candidate variables hypothesized to govern repair status, including infection intensity at 3 dpi, sex, and donor age. Details of this index are shown in **Supplemental File 9.**

J. Relationship of 14 dpi repair status on long pulmonary COVID syndrome

A list of DEGs was generated from comparisons of 14 dpi vs mock cultures, and this list of genes was made into a "repair status" pathway (the 14dpi *in vitro* DEG signature). GSEA analyses were then used to test for enrichment of expression of the 14 dpi *in vitro* DEG signature in samples from three long pulmonary COVID human subject cohorts. First, we analyzed a published spatial transcriptomics dataset of COVID autopsy lungs with a prolonged clinical course (> 20 d) and pulmonary fibrotic changes, focusing specifically on the epithelial cell gene signatures from bronchioles and microcysts regions from this larger dataset (23). Details are provided in **Supplemental Files 10 and 11**.

Second, we studied a cohort of five lung biopsies obtained by visually assisted trans-thoracoscopic (VATS) procedures from subjects 9-12 months post documented SARS-CoV-2 infection with persistent CT abnormalities consistent with focal fibrotic changes. GeoMx spatial transcriptomic studies were performed on formalin-fixed paraffin-embedded (FFPE) blocks generated from these biopsies cut into 5 µm sections. Regions of interest were placed on bronchioles and MUC5B mucin containing microcysts as determined from H&E-stained sections and MUC5B immunohistochemistry. Data were analyzed as previously published (23, 24). Raw count, and upper quartile (Q3) normalized data were exported from NanoString DSP (Digital Spatial Profile) server. Differential gene expression analyses from raw count data were performed using the dream function from the Bioconductor R package, variancePartition (12) to

account for related ROIs from the same lung and assay slides as random-effect factors. Pre-ranked gene set enrichment analysis (GSEA) was performed using the Bioconductor R package, fgsea (19), with gene set collections obtained from Reactome pathways (20). Gene expression values from the GeoMX dataset used in this manuscript are provided in **Supplemental File 12**.

Third, we studied single-cell RNAseq datasets generated from airway epithelia obtained by bronchial brushes in subjects with persistent long-COVID airways disease manifested by respiratory symptoms (cough and sputum) ~ 18 mo post documented SARS-CoV-2 infection (17). Pathway analyses of this published dataset is provided in **Supplemental File 13**.

K. Statistical considerations

Differences of means of outcome measurements between experimental groups were tested with linear models (*lm* from base R), or in the case of repeated measures design, with linear mixed-effect models (*lmer* from R package, *lme4* (25) and lmerTest (26), treating donor code as a random-effect factor. For specific associations between dependent variables (*e.g.*, viral RNA – log transformed TPM as y) and an independent variable (*e.g.*, sex as x), 3 levels of stats and p-values were reported: a) univariable correlation between y~x; b) y~x plus multiple independent variables as biological co-variates, age group, time, degree of ciliation (median of ciliated genes as surrogate); and c) with additional technical and QC variables, batch and PC#1. The significance at each model level were denoted as *, †, and ‡. The linear mixed-effect models for bulk RNA-seq and proteomic data included all the appropriate biological and technical co-variates.

References

1. Hou YJ, et al. SARS-CoV-2 D614G variant exhibits efficient replication ex vivo and transmission in vivo. *Science.* 2020;370(6523):1464-1468.
2. Hou YJ, et al. SARS-CoV-2 Reverse Genetics Reveals a Variable Infection Gradient in the Respiratory Tract. *Cell.* 2020;182(2):429-446.e414.
3. Okuda K, et al. Localization of Secretory Mucins MUC5AC and MUC5B in Normal/Healthy Human Airways. *Am J Respir Crit Care Med.* 2019;199(6):715-727.
4. McElvaney OF, et al. Protease-anti-protease compartmentalization in SARS-CoV-2 ARDS: Therapeutic implications. *EBioMedicine.* 2022;77:103894.
5. Wisniewski JR, et al. Universal sample preparation method for proteome analysis. *Nat Methods.* 2009;6(5):359-362.
6. Kesimer M, et al. Excess Secretion of Gel-Forming Mucins and Associated Innate Defense Proteins with Defective Mucin Un-Packaging Underpin Gallbladder Mucocele Formation in Dogs. *PLoS One.* 2015;10(9):e0138988.
7. Perkins DN, et al. Probability-based protein identification by searching sequence databases using mass spectrometry data. *Electrophoresis.* 1999;20(18):3551-3567.
8. Nesvizhskii AI, et al. A statistical model for identifying proteins by tandem mass spectrometry. *Anal Chem.* 2003;75(17):4646-4658.
9. Rainer J, et al. A Modular and Expandable Ecosystem for Metabolomics Data Annotation in R. *Metabolites.* 2022;12(2).
10. Dobin A, et al. STAR: ultrafast universal RNA-seq aligner. *Bioinformatics.* 2013;29(1):15-21.
11. Pertea M, et al. Transcript-level expression analysis of RNA-seq experiments with HISAT, StringTie and Ballgown. *Nat Protoc.* 2016;11(9):1650-1667.

12. Hoffman GE, Roussos P. Dream: powerful differential expression analysis for repeated measures designs. *Bioinformatics.* 2021;37(2):192-201.

13. Okuda K, et al. Secretory Cells Dominate Airway CFTR Expression and Function in Human Airway Superficial Epithelia. *Am J Respir Crit Care Med.* 2021;203(10):1275-1289.

14. Thioulouse J, et al. *Multivariate analysis of ecological data with ade4.* New York: Springer; 2018.

15. Gu Z, et al. Complex heatmaps reveal patterns and correlations in multidimensional genomic data. *Bioinformatics.* 2016;32(18):2847-2849.

16. Wickham H. *ggplot2: Elegant Graphics for Data Analysis.* New York: Springer-Verlag; 2016.

17. Gerayeli FV, et al. Single-cell sequencing reveals cellular landscape alterations in the airway mucosa of patients with pulmonary long COVID. *Eur Respir J.* 2024;64(5).

18. He L, et al. NEBULA is a fast negative binomial mixed model for differential or co-expression analysis of large-scale multi-subject single-cell data. *Commun Biol.* 2021;4(1):629.

19. Korotkevich G, et al. Fast gene set enrichment analysis. *bioRxiv.* 2021:060012.

20. Grentner A, et al. ReactomeGSA: new features to simplify public data reuse. *Bioinformatics.* 2024;40(6).

21. Esther CR, Jr., et al. Sialic acid-to-urea ratio as a measure of airway surface hydration. *Am J Physiol Lung Cell Mol Physiol.* 2017;312(3):L398-L404.

22. Esther CR, Jr., et al. Metabolomic biomarkers predictive of early structural lung disease in cystic fibrosis. *Eur Respir J.* 2016;48(6):1612-1621.

23. Kato T, et al. Prevalence and Mechanisms of Mucus Accumulation in COVID-19 Lung Disease. *Am J Respir Crit Care Med.* 2022;206(11):1336-1352.

24. Dinnon KH, 3rd, et al. SARS-CoV-2 infection produces chronic pulmonary epithelial and immune cell dysfunction with fibrosis in mice. *Sci Transl Med.* 2022;14(664):eabo5070.

25. Bates D, et al. Fitting Linear Mixed-Effects Models Using lme4. *J Stat Softw.* 2015;67(1):1 - 48.

26. Kuznetsova A, et al. lmerTest Package: Tests in Linear Mixed Effects Models. *J Stat Softw.* 2017;82(13):1 - 26.

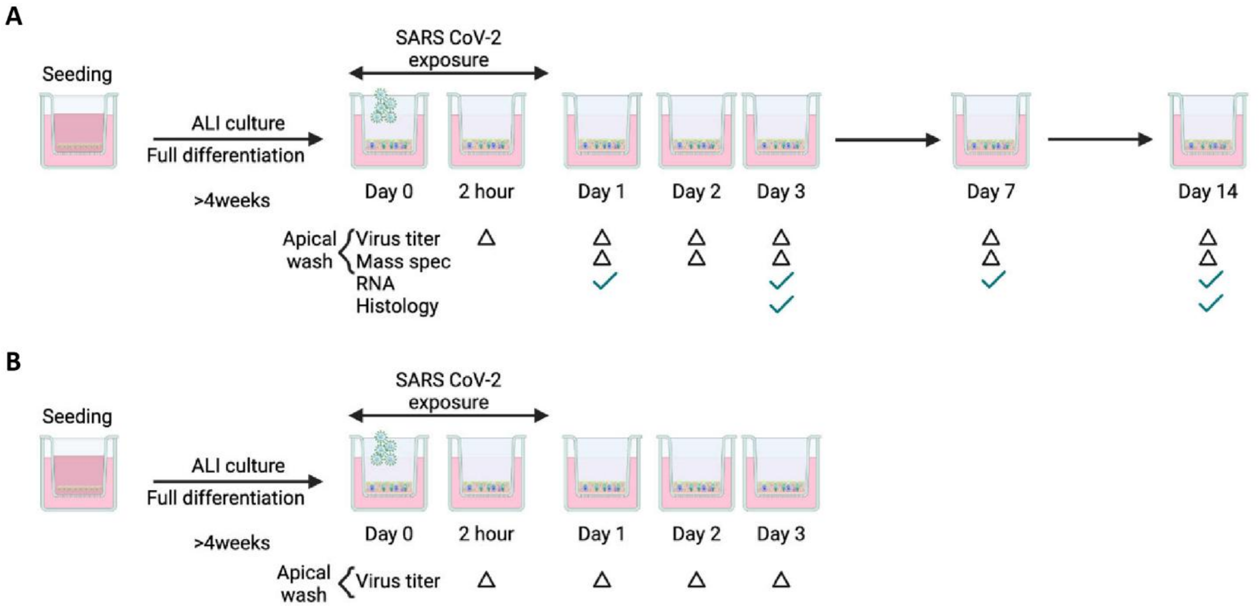

Supplemental Figure 1. Diagram of protocol/sampling sequences for SARS-CoV-2-infected vs. mock-exposed human bronchial epithelial (HBE) cells. (A) Protocol 1, which occurred in two batches and extended 14 days post infection (dpi). (B) Protocol 2, in which all samples were grown and infected as one batch, with the study ending at 3 dpi. For each of the protocols, freshly isolated passage 2 HBE cells were seeded onto Millicell inserts and studied after full differentiation (> 28d) under air–liquid interface (ALI) conditions. D614G SARS-CoV-2 inoculation was performed for 20 min at d0. Mock cultures were subjected to a 20 min inoculation with PBS. Apical surface samples for SARS-CoV-2 titers were obtained immediately after post-inoculation washes (2 hr) and at timepoints as indicated with open triangles. For Protocol 1 (A) only, aliquots of apical wash samples at 1, 2, 3, 7, and 14 dpi were provided for mass spectroscopy (mass spec) protein analyses, SARS-CoV-2 and mock inoculated HBE cultures were subjected to RNA harvesting at 1, 3, 7, and 14 dpi, and mock and SARS-CoV-2 inoculated HBE cultures were fixed and submitted for histologic evaluation 3 and 14 dpi. Protocol 2 was utilized specifically for titer only.

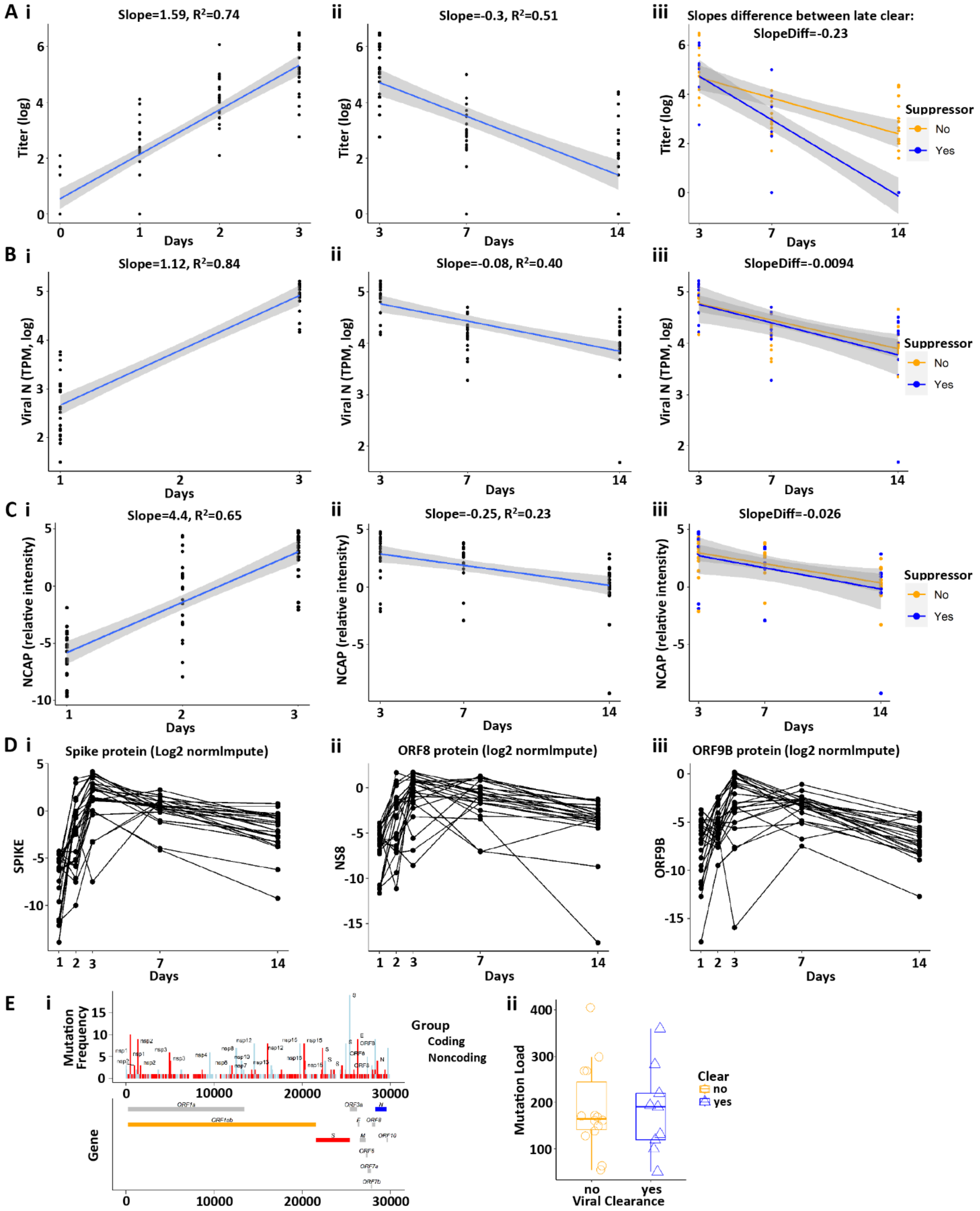

Supplemental Figure 2. SARS-CoV-2 infection of HBE cultures: SARS-CoV-2 titer, TPM, and protein indices. (A) Rates of acquisition and suppression of SARS-CoV-2 titers in HBE cultures post-inoculation. **(Ai)** Rate of acquisition of SARS-CoV-2 titerable virus from 0-3 dpi (slope = 1.59, SE 0.098, $r^2 = 0.74$, p = < 0.001). **(Aii)** Rate of SARS-CoV-2 titerable virus reduction from 3 to 14 dpi (slope = -0.3,

SE 0.035, $r^2 = 0.51$, p = < 0.001). **(Aiii)** Rates of viral suppression for suppressors (blue) vs. non-suppressors (yellow); slopes different (-0.23 SE 0.057) $p < 0.01$. Slope for suppressor group, -0.444 (SE 0.045), non-suppressor slope, -0.21 (SE 0.034) **(B)** Acquisition vs. suppression rates for intracellular SARS-CoV-2 N transcripts. **(Bi)** Rate of acquisition from 1 and 3 dpi sample (slope = 1.12 (SE 0.072), $r^2 = 0.54$, $p < 0.001$). **(Bii)** Rate of suppression from 3, 7, and 14 dpi sample (slope = -0.08 SE 0.012, $r^2 = 0.40$, $p < 0.001$). **(Biii)** Suppression rates for SARS-CoV-2 titer suppressors (blue) vs. non-suppressors (yellow); slope difference = non-significant. **(C)** Acquisition vs. suppression rates for NCAP proteins in SARS-CoV-2-infected HBE cultures. **(Ci)** Rate of acquisition from all 1, 2, and 3 dpi HBE culture samples (slope = 4.4 SE 0.39, $p < 0.001$). **(Cii)** Rate of suppression for all HBE cultures over 3, 7, and 14 dpi (slope = -0.25, p = 0.128). **(Ciii)** Rate of suppression for SARS-CoV-2 titer suppressors (blue) vs. non-suppressors (yellow); slope difference = non-significant. **(D)** SARS-CoV-2 protein levels on HBE culture surfaces post-infection: **(Di)** Spike protein; **(Dii)** ORF8; **(Diii)** ORF9B. **(E)** Mutation analyses within SARS-CoV-2 genome showing **(Ei)** locations of identified mutations across the length of the viral genome. **(Eii)** The mutation load did not differ between suppressor and non-suppressor cultures.

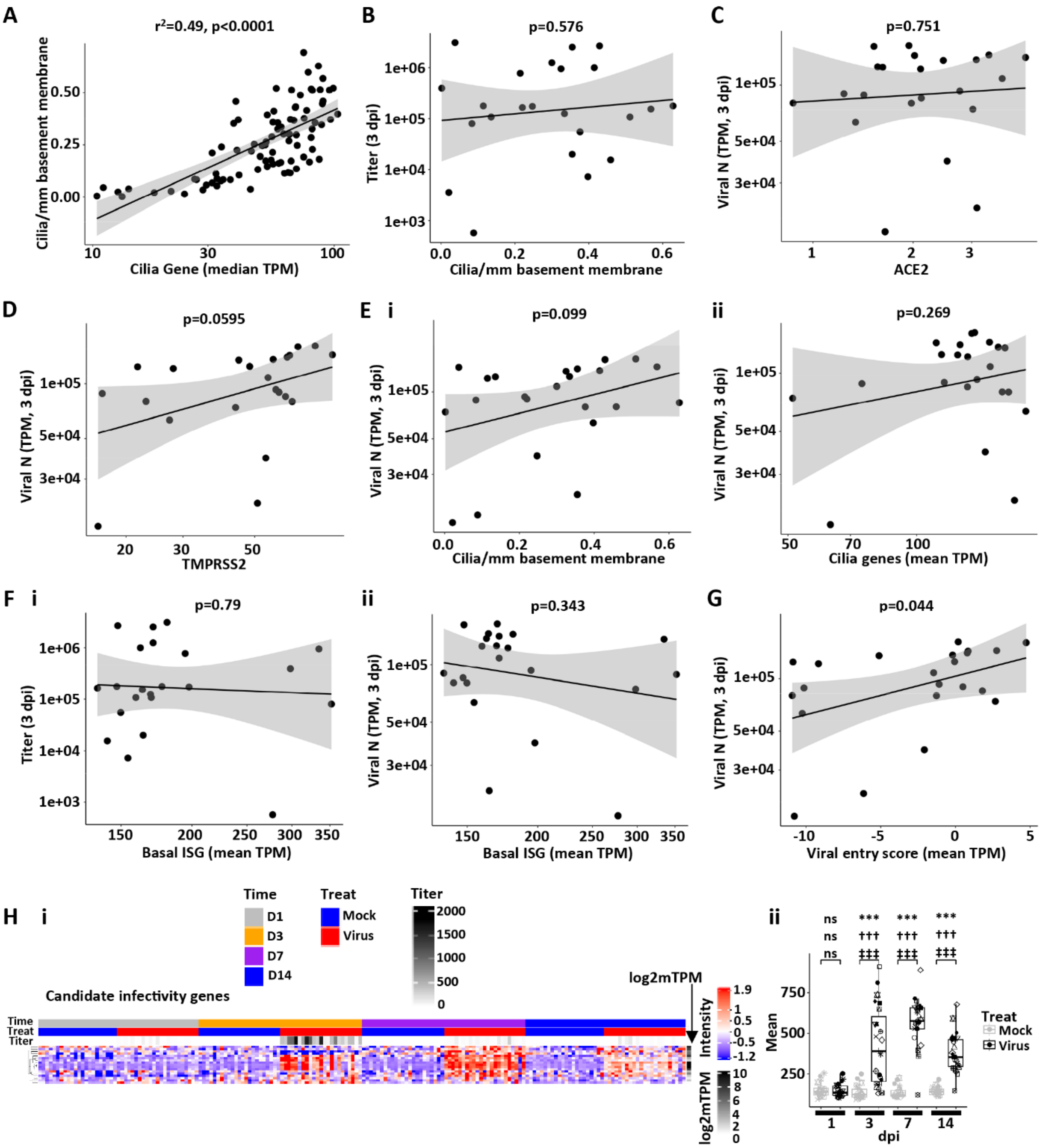

Supplemental Figure 3. Human bronchial epithelial host defense properties as related to SARS-CoV-2 infectivity. (A) Relationships between histologic measure of cell ciliation (number of ciliated cells/basement membrane length, nCilia.BM) vs. median ciliated cell gene expression set ($r^2 = 0.49$, $p < 0.001$, slope=0.16, SE 0.017). **(B)** Relationship between nCilia.BM and 3 dpi titer. **(C)** Full-length ACE2 RNA expression in mock d1 HBE cultures vs. peak intracellular viral N transcript expression at 3 dpi ($r^2 = 0.03$, $p = 0.751$). **(D)** TMPRSS2 RNA expression in mock 1 dpi HBE culture vs. 3 dpi culture SARS-CoV-2 N transcript abundance ($r = 0.17$, $p = 0.0595$, slope=0.15, SE 0.076). **(E)** Indices of HBE culture ciliation vs. SARS-CoV-2 transcript levels. **(Ei)** Histologic measure of ciliation (nCilia.BM) vs. SARS-CoV-2 N viral transcript abundance at 3 dpi ($r^2 = 0.124$, $p = 0.099$). **(Eii)** Ciliated gene expression mean in mock d1 HBE culture vs. SARS-CoV-2 N transcript abundance at 3 dpi ($r^2 = 0.054$, $p < 0.269$,

slope=0.13, SE 0.12). **(F)** Baseline interferon signaling and infection. **(Fi)** Baseline ISG RNA transcript levels in mock d1 HBE cultures vs. peak SARS-CoV-2 titer at 3dpi. $r^2 = 0.0036$, $p = 0.79$. **(Fii)** Baseline ISG RNA transcript levels in mock d1 HBE cultures vs. SARS-CoV-2 N transcript levels at 3 dpi ($r^2 = 0.045$, $p = 0.343$) **(G)** Viral entry score vs. peak SARS-CoV-2 N TPM. $r^2 = 0.187$, $p = 0.044$ (slope=0.022, SE 0.01). **(H)** Candidate viral infectivity genes. **(Hi)** Heatmap depicting candidate viral infectivity gene expression in mock and SARS-CoV-2-infected cultures over time post-infection. **(Hii)** Box plots depicting candidate infectivity genes in individual HBE culture donors for mock and SARS-CoV-2-infected cultures over time post infection. For Hii, statistics performed, and symbol definitions defined as in main text, legend to **Figure 3.**

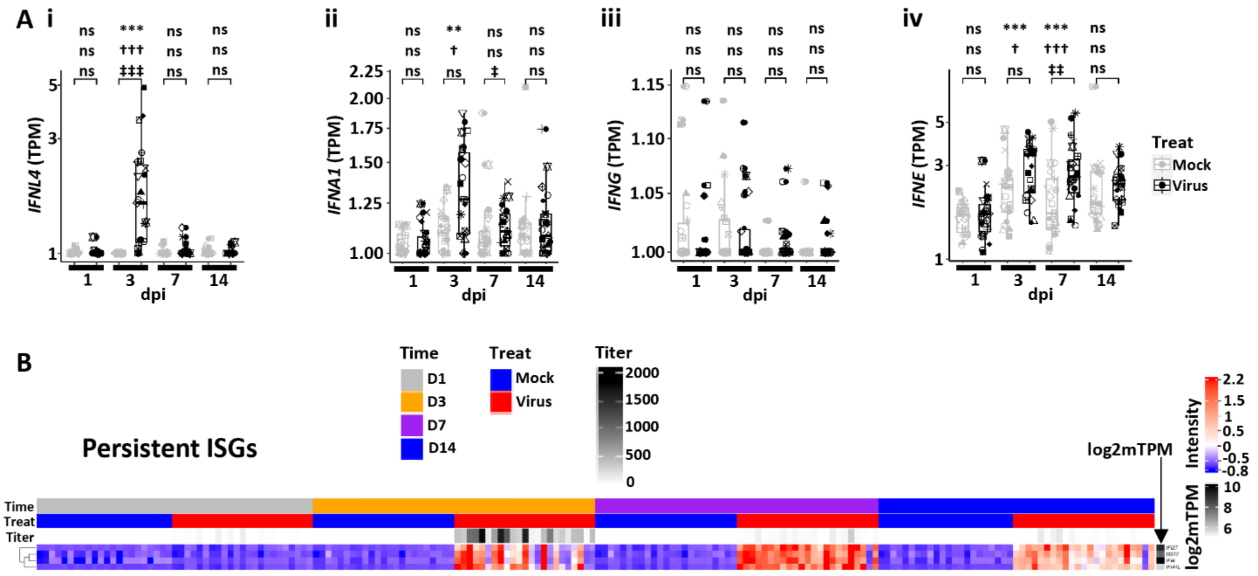

Supplemental Figure 4. Interferon and interferon-stimulated gene (ISG) responses post-SARS-CoV-2 HBE culture infection. **(A)** Interferon gene RNA expression in mock and SARS-CoV-2-infected HBE cultures as a function of time post-infection. **(Ai)** IFNL4. **(Aii)** IFNA1. **(Aiii)** IFNG. **(Aiv)** IFNE. **(B)** Heatmap of ISGs with higher fold changes than mock samples at 14 dpi than at 3 dpi, which were termed "persistent" ISGs.

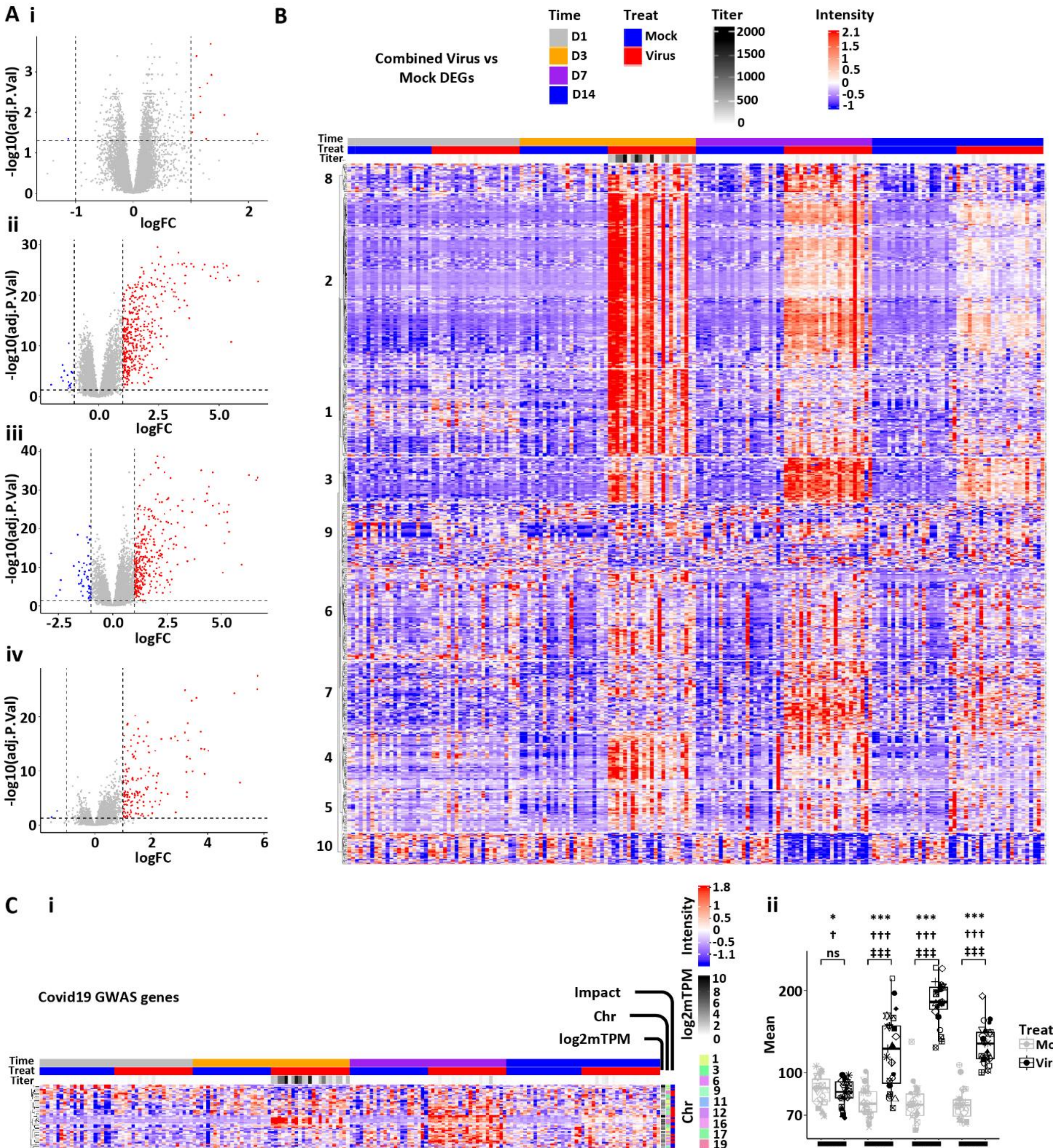

Supplemental Figure 5. Global epithelial responses to SARS-CoV-2. **(A)** Volcano plots depict DEGs for the comparison mock vs. SARS-CoV-2 infection at **(Ai)** 1 dpi; **(Aii)** 3 dpi; **(Aiii)** 7 dpi; and **(Aiv)** 14 dpi. Red indicates genes upregulated by virus; blue indicates genes downregulated by virus. DEGs represented are filtered at adjusted P-value of <0.05 (horizontal dotted lines) and absolute log2 fold-change >1 (vertical dotted lines) with an average expression >0 (log2; or roughly TMP>1). **(B)** Combined heatmap of DEGs representing the comparison SARS-CoV-2-infected vs. mock for all dpi listed. Top bars indicate major treatment groupings with key provided to the left of the heatmap. **(C)** Kinetics of GWAS associated genes. **(Ci)** Heatmap showing pattern of expression of genes identified in GWAS studies

associated with COVID-19 clinical features. **(Cii)** Boxplot representing the mean response of all genes represented in the heatmap across dpi in mock and infected cultures.

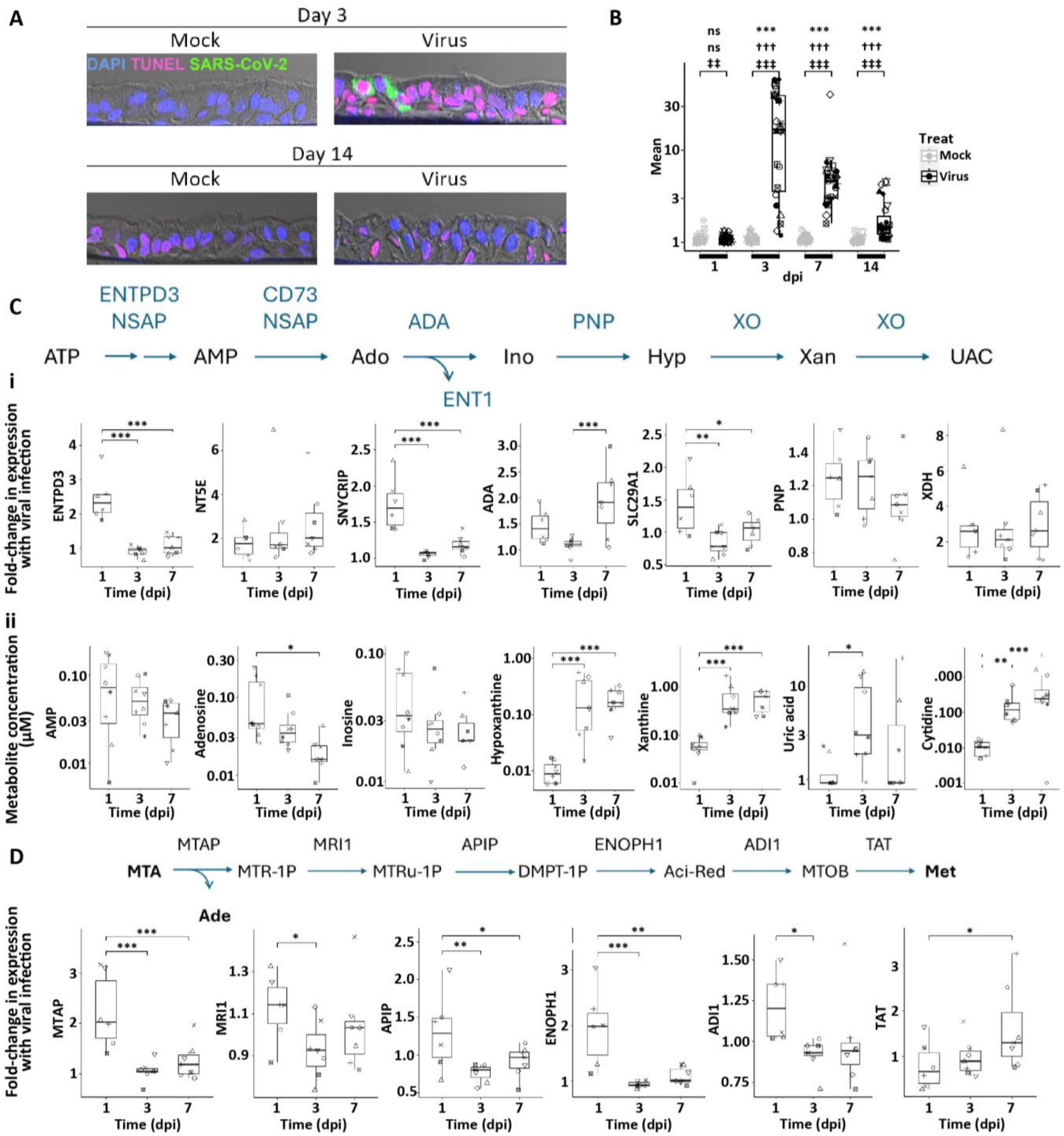

Supplemental Figure 6. HBE cell responses at time of peak SARS-CoV-2 infection. (A) Immunocytochemistry of mock vs. SARS-CoV-2-infected HBE cultures at 3 and 14 dpi for SARS-CoV-2 N capsid protein (green), TUNEL (chartreuse), and DAPI (blue). **(B)** Expression of ZBP1 as a function of time and viral infection. **(C)** Extracellular enzymes that metabolize ATP released onto HBE culture surfaces and ATP metabolites. **(Ci)** Adenylyl purine nucleotides and metabolic products on mock vs. SARS-CoV-2-infected HBE culture surfaces at 1, 3, and 7 dpi. AMP = adenosine monophosphate; Ado = adenosine; Ino = inosine; Hyp = hypoxanthine; Xan = xanthine; UAC = uric acid; Cyt = cytosine. **(Cii)** HBE culture RNA expression for extracellular enzymes that metabolize released ATP to cytosine on airway surfaces at 1, 3, and 7d post-SARS-CoV-2 infection. ENTPD3 = ectonucleoside triphosphate

diphosphohydrolase- 3; CD73; NSAP = non-specific alkaline phosphatase; ADA = adenosine deaminase; ENT1 = solute carrier family 29 member 1; PNP = purine nucleoside phosphorylase; XO = xanthine oxidase. *$p < 0.05$; **$p < 0.01$; ***$p < 0.001$; ****$p < 0.0001$. **(D)** Methionine salvage pathway. Enzymes that metabolize methyl-thio-adenosine to methionine are shown. In the bar graphs, the fold change in extracellular methionine salvage pathway enzymes in SARS-CoV-2-infected cultures at 1, 3, and 7 dpi are indicated. **$p < 0.01$; ***$p < 0.001$; ****$p < 0.0001$.

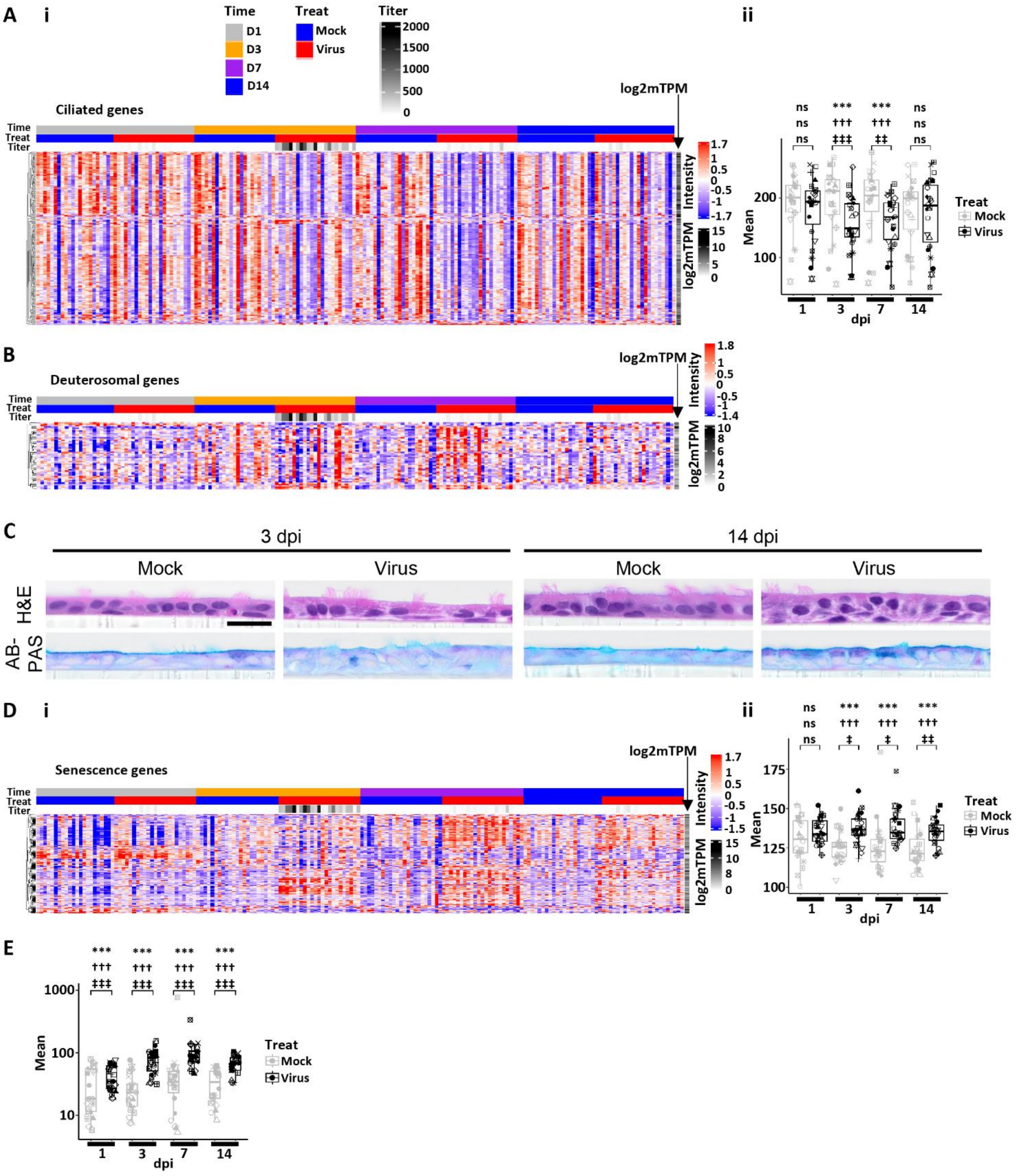

Supplemental Figure 7. HBE remodeling, senescence, inflammation, and repair post-SARS-CoV-2 infection. **(A)** Ciliated cell gene responses to SARS-CoV-2 infection. **(Ai)** Heatmap depicting ciliated gene expression over dpi. **(Aii)** Box plots depicting mean ciliated gene expression over time dpi. **(B)** Heatmap depicting deuterosomal genes over time dpi. **(C)** Representative H&E and AP-PAS histologic stains of mock and SARS-CoV-2-infected HBE cultures at 3 and 14 dpi. **(D)** Senescence gene responses. **(Di)** Heatmap depicting senescence gene expression over time dpi. **(Dii)** Box plots depicting mean

senescence gene expression with time dpi. Statistics performed, and symbol definitions defined, as in main text, legend to **Figure 3.**

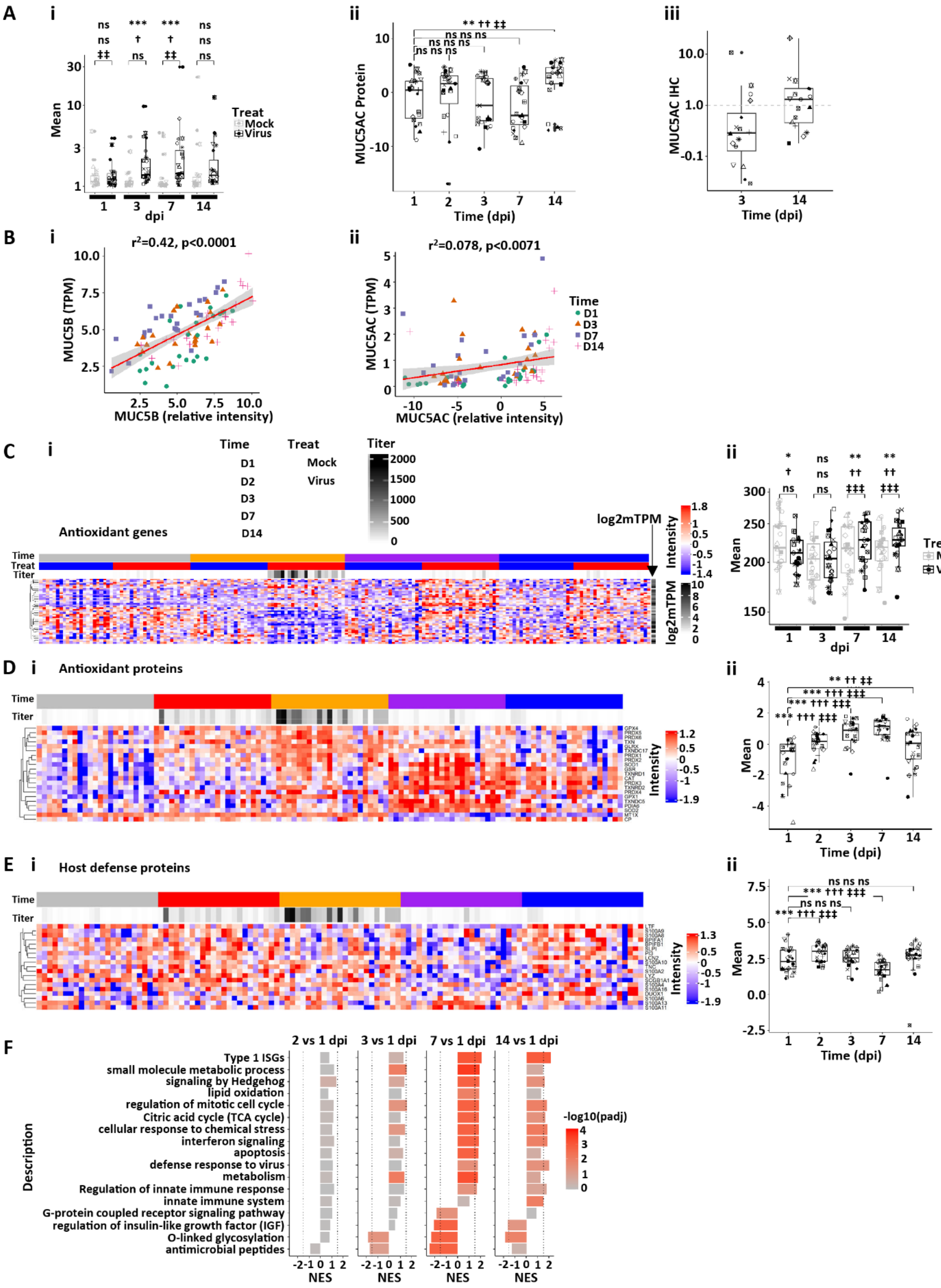

Supplemental Figure 8. Human bronchial epithelial host defense responses to SARS-CoV-2 infection. (A) MUC5AC RNA and protein expression. **(Ai)** Relationship between SARS-CoV-2-infected

HBE culture MUC5AC RNA expression (log2/MUC5AC+1) and **(Aii)** MUC5AC protein on HBE culture surfaces with time post-SARS-CoV-2 infection. **(Aiii)** Fold change of MUC5AC on histological sections at 3 and 14 dpi as evaluated by immunohistochemistry (not significant). **(B)** Secreted mucin protein levels vs. gene expression levels for **(Bi)** MUC5B ($R^2 = 0.42$, $p<0.0001$, slope=0.51, SE 0.063) and **(Bii)** MUC5AC ($R^2 = 0.078$; $p = 0.0071$, slope=0.05, SE 0.018). **(C)** Antioxidant genes. **(Ci)** Heatmap depicting mean antioxidant gene expression in mock vs. SARS-CoV-2-infected HBE cultures with time. **(Cii)** Mean antioxidant gene expression with time dpi. **(D)** Antioxidant proteins. **(Di)** Heatmap depicting levels of antioxidant proteins over time. **(Dii)** Mean antioxidant protein levels over time dpi. **(E)** Host defense proteins. **(Ei)** Heatmap depicting host defense proteins on surfaces over time dpi. **(Eii)** Mean host defense protein levels over time dpi. **(F)** Normalized enrichment score (NES) for selected Reactome + custom pathway annotations resulting from analyses of differentially expressed secreted protein levels for the comparisons indicated (all comparisons are compared to dpi 1, which is considered baseline for this analysis). The intensity of the red color indicates the significance of the pathway enrichment at each dpi comparison. Only pathways significant for at least one comparison (significance represented by vertical dotted line) are shown. Negative values for NES indicated that the annotated pathway is downregulated in virus treated samples compared to dpi 1 samples; positive NES values indicate upregulation.

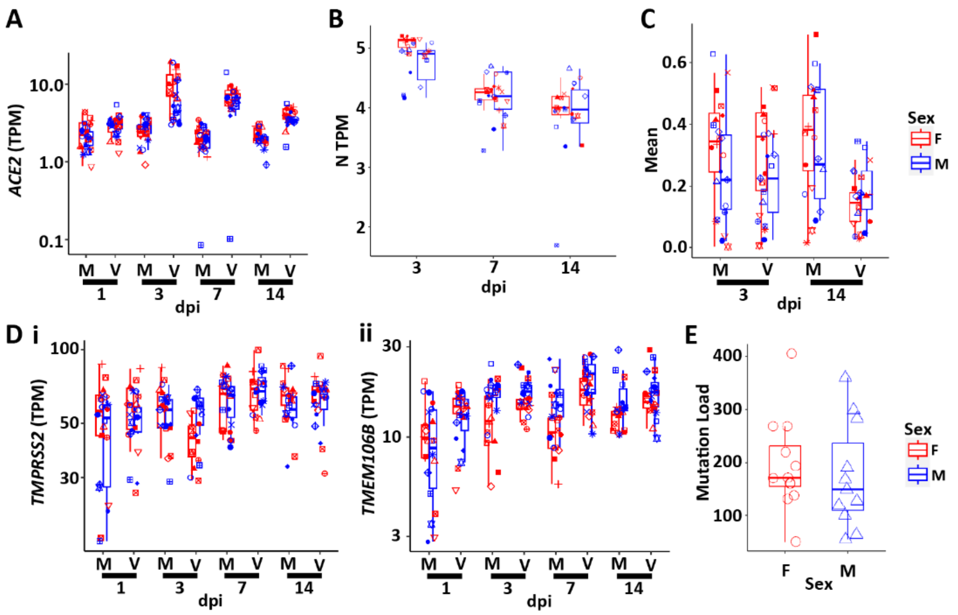

Supplemental Figure 9. SARS-CoV-2 infectivity and HBE responses as a function of sex. (A) Box plots depicting ACE2 transcript levels in female vs. male HBE cultures over 1-14 dpi. **(B)** Box plots depicting viral transcripts in female vs. male HBE cultures over 3-14 dpi. **(C)** Ciliated cell abundance per basement membrane length (nCilia.BM) for the treatments shown separated by sex. No significant difference is found between sexes at any timepoint. **(D)** Host factors. Expression of **(Di)** TMPRSS2 and **(Dii)** TMEM106B for the treatment categories listed separated by sex. **(E)** SARS-CoV-2 mutation loads at 14 dpi for female vs. male HBE cultures. No significant differences were found between male and female at any timepoint tested.

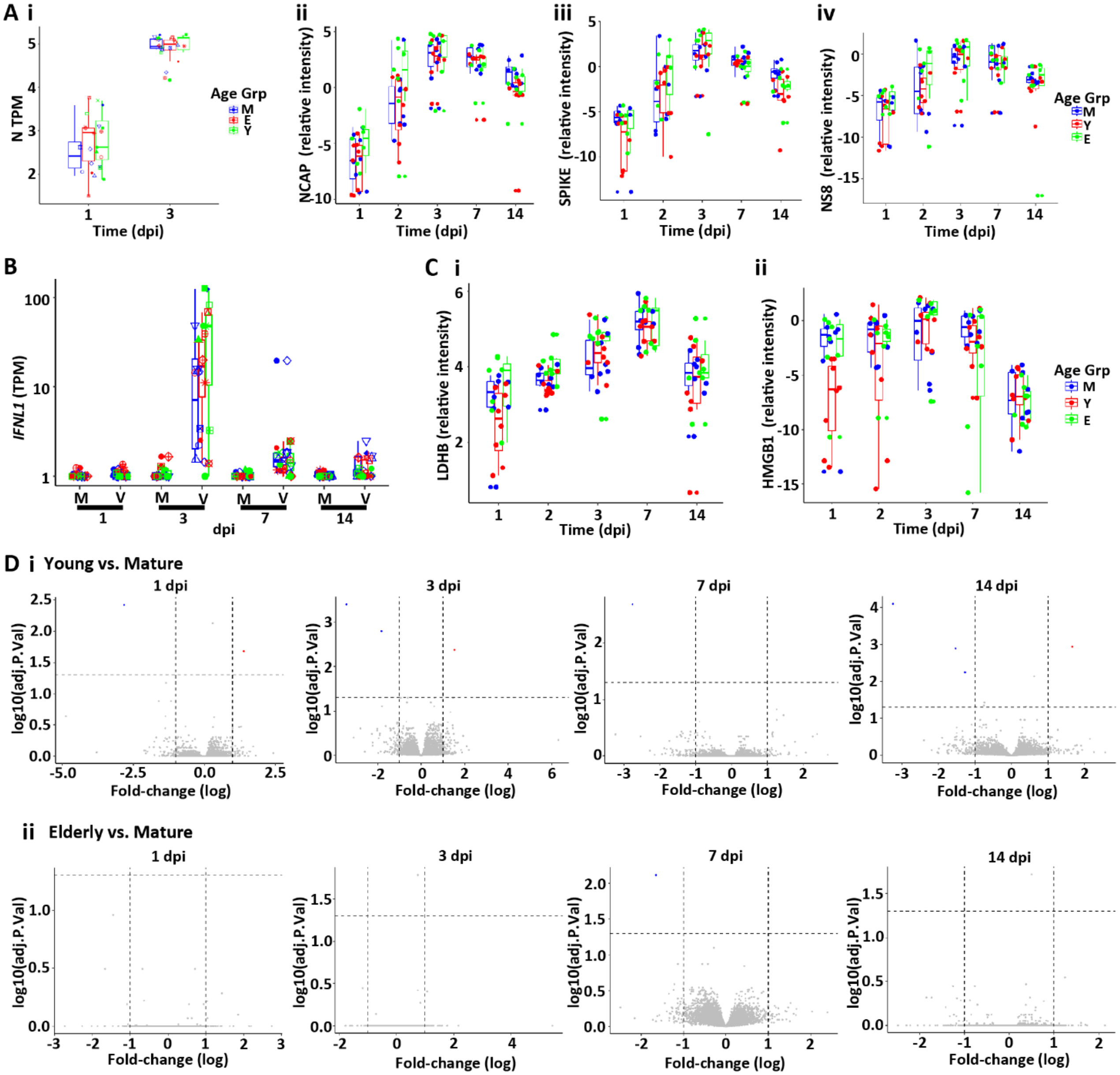

E

Suppressor	Young	Mature	Elderly
No	3	6	5
Yes	4	2	3

F

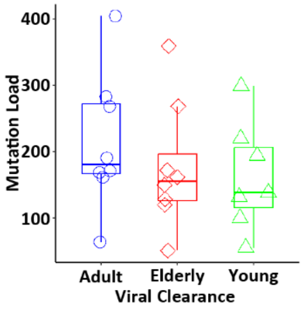

Supplemental Figure 10. Infectivity and response as a function of age. (A) Age related responses. **(Ai)** Infectivity from 1 and 3 dpi as a function of age and viral transcripts as represented by viral N TPM. **(Aii)** SARS-CoV-2 protein levels in apical secretions for each dpi shown colored by age for NCAP; **(Aiii)** Spike; **(Aiv)** ORF8. **(B)** INFL1 expression for mock and SARS-CoV-2-infected cultures colored by age

with time dpi. For A and B, no significant differences were found among age group comparisons at all timepoints tested. **(C)** Damage-associated secreted protein levels for **(Ci)** LDHB and **(Cii)** HMGB1for dpi as shown and shown for each age group with time dpi. **(D)** Volcano plots showing DEGs in Young vs. Mature and Elderly vs. Mature in virus-treated cultures for the dpi indicated. Red indicates genes that are significantly upregulated; blue indicates genes significantly downregulated. DEGs represented are filtered at adjusted P-value of <0.05 (horizontal dotted lines) and absolute log2 fold-change >1 (vertical dotted lines) with an average expression >0 (log2; or roughly TMP>1). NOTE: the number of DEGs is very limited, with no DEGs for Elderly vs. Mature. (**E)** Table indicating the distribution of suppressors and non-suppressors across ages. The results are not significant (chi-square test p-value = 0.44) but a higher percentage of young cultures are to be suppressors (57%) compared to mature (25%) and elderly (38%). **(F)** The determined mutation load distributed by age. There were no significant differences in mutation load across ages.

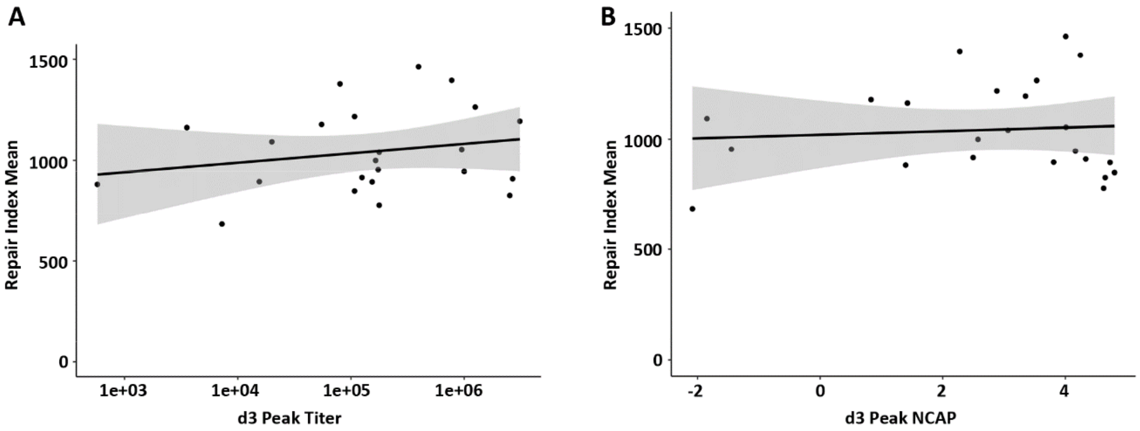

Supplemental Figure 11. Relationships of the repair index at 14 dpi to **(A)** mean peak titer (slope=0.067, SE 0.064, r2=0.049, p=0.311) and **(B)** peak secreted NCAP protein (slope=0.012, SE 0.03, r2=0.0077, p=0.691). No significant correlations could be identified.
