# Supplementary material for: Airway Epithelial SARS-CoV-2 Infectious and Repair Responses: Relationships to Age, Sex, and Post-COVID Pulmonary Syndromes
Source: bioRxiv. 2025 Jul 17:2025.07.17.663733. Preprint. [Version 1] doi: 10.1101/2025.07.17.663733 (PMC12338744; doi:10.1101/2025.07.17.663733)
Supplement: Supplementary file 2 [file media-3.pdf]

| **Supplemental Table 2.** Details of Supplemental Files that accompany the manuscript as separate files. Please note that the column headings for each file are considered self-explanatory, but any questions related to them should be directed to the authors. | | |
|---|---|---|
| **File Number** | **File Name** | **File Description** |
| Supplemental File 1 | Sample information and viral titers for Protocol 1 | Contains details of individual samples and treatments related to Protocol 1 bulk RNAsequencing data. Since the experiment was conducted in two batches, these batches are important in analyses and are provided. The file also contains the results of viral titers, whether the samples were classified as suppressor cultures, individual transcripts per million (TPM) for viral genes, the calculated value for cilia features from gene expression as described in the methods, the measurement of cilia/basement membrane as described in the methods, and principal component 1. The table provides the necessary data for replication of the analyses presented throughout the paper, which utilize demographic variables as well as batch, ciliation, and PCs as covariates to control for technical variation (batch, PC1) and a variable known to affect viral infectivity (ciliation). |
| Supplemental File 2 | Titers measured for Protocol 2 (replication study) | Raw titer data for Protocol 2; not this protocol extended only to 3 dpi |
| Supplemental File 3 | Virus mutations identified in 14 dpi culture secretions | Contains individual mutation data identified as described in methods for each culture sample associated with 14 dpi secretions in Protocol 1. The first worksheet provides the mutation calls, and the second provides the calculated mutation load. |
| Supplemental File 4 | Raw data for TUNEL quantification | Provides the raw values for TUNEL staining. |
| Supplemental File 5 | Data from secreted proteome | Provides the individual sample data for the proteomics analyses as described in the methods. The three worksheets provide the necessary raw data and sample information to reproduce the figures presented in the manuscript; Worksheet 1 provides sample information and virus proteins; Worksheet 2 provides raw protein measurements for all samples and all proteins; Worksheet 3 provides the values utilized in the analyses for the paper, which utilized filtering and imputation as described. |
| Supplemental File 6 | Gene lists for heat maps presented | The genes associated with each heat map presented in the manuscript are listed. These lists are provided because the genes themselves are not readable in the figure files due to size constraints. The list of genes provided is a subset of the original pathways due to the filtering criteria applied as described in the methods. |

| | | |
|---|---|---|
| Supplemental File 7 | Gene lists for custom gene sets | We have generated gene sets based on published literature and individual expert knowledge that we have added as pathways to the REACTOME pathways. These are often significant in our GSEA analyses. The genes in these pathways are provided in this file (Worksheet 1 = geneSets) and the sources are provided in Worksheet 2 = geneSetNames. |
| Supplemental File 8 | Metabolomic data | This file provides the individual data values used to support Supplemental Figure 6 C and D. |
| Supplemental File 9 | Details of gene set of repair genes | This file provides a list of genes that was used from each pathway to define the gene repair index utilized as described in the methods for Figure 10. |
| Supplemental File 10 | GeoMx gene level expression data from long Covid autopsy | This file provides information related to the GeoMX spatial transcriptomics data that was used to analyze the long Covid autopsy specimens described in Figure 10. |
| Supplemental File 11 | Differential gene expression for long Covid autopsy | This table provides analyses of differential gene expression of the long Covid autopsy GeoMx data associated with Figure 10. Covariates used in the model included: batch, PC1, and donor, GeoMx assay slide as random effects. |
| Supplemental File 12 | Pathway analyses for long Covid autopsy | This table provides pathway analysis for the Covid autopsy specimens described in Figure 10. |
| Supplemental File 13 | GeoMx gene level expression data from long Covid biopsy | This table provides the information related to the GeoMX spatial transcriptomics data that was used to analyze the long Covid biopsy specimens described in Figure 10. |
| Supplemental File 14 | Differential gene expression for long Covid biopsy | This table provides the analyses of differential gene expression of the long Covid biopsy GeoMx data associated with Figure 10. Covariates used in the model included: batch, and donor,GeoMx assay slide as random effects. |
| Supplemental File 15 | Pathway analyses for long Covid biopsy | This table provides the pathway analyses for the Covid biopsy specimens described in Figure 10. |
| Supplemental File 16 | Differential gene expression for long Covid scRNA from bronchial brushes | This table provides fold change values for differential gene expression between long Covid and controls from the scRNAsequencing data as described in the methods associated with the bronchial brushings in Figure 10. Covariates used in the model included: age, gender. |
| Supplemental File 17 | Pathway analyses for long Covid bronchial brushes | This table provides pathway analyses of scRNAsequencing data as described in the methods associated with bronchial biopsies in long Covid in Figure 10. |
| Supplemental File 18 | Differential gene expression of virus vs mock across time (dpi) | This file provides the results of differential gene expression for virus versus mock for all time points post-infection for Protocol 1. Covariates used in the model included: age group, sex, batch, PC1, median TPM of 363 cilia cell marker genes, and donor as random effect. |

| | | |
|---|---|---|
| Supplemental File 19 | GSEA pathway analyses for Virus vs Mock across time (dpi) | This file provides pathways results for virus vs mock for all time points post-infection. The pathways listed are a combination of REACTOME pathways and our custom pathways. |
| Supplemental File 20 | Differential gene expression of male vs female across time (dpi) MockOnly | This file providetime pointslts of differential gene expression for male vs female in mock infected cultures for all time points post-infection for Protocol 1. Covariates used in the model included: age group, batch, PC1, median TPM of 363 cilia cell marker genes, and donor as random effect. |
| Supplemental File 21 | GSEA pathway analyses for Male vs Female across time (dpi)_time MockOnly | This file provides pathways results for male vs female for all timepoints in mock infected cultures only. The pathways listed are a combination of REACTOME pathways and our custom pathways. |
| Supplemental File 22 | Differential gene expression of Young vs Mature and Elderly vs Mature (all comparions) | This file provides the results of differential gene expression for comparisons across the age groups for all timepoints post-infection for Protocol 1. The file includes comparisons for Young vs Mature and Elderly vs Mature for both mock treated and virus treated cultures across all timepoints tested in Protocol 1. Covariate used in the model included: sex, batch, PC1, median TPM of 363 cilia cell marker genes, and donor as random effect. |
| Supplemental File 23 | GSEA pathway analyses for Young vs Mature and Elderly vs Mature (all comparisons) | This file provides pathways results comparisons across the age groups for all timepoints post-infection for Protocol 1. The file includes comparisons for Young vs Mature and Elderly vs Mature for both mock treated and virus treated cultures across all timepoints tested in Protocol 1. The pathways listed are a combination of REACTOME pathways and our custom pathways. |
| Supplemental File 24 | Differential expression of secreted proteome 1 dpi compared to 3, 7, 14 dpi | This file provides results of differential secreted protein expression measured by proteomics. The comparisons are 1 dpi secreted proteins vs 3, 7, and 14 dpi secreted proteins. Covariates included in the model included sex, age group, suppressor status, and donor as random effect. |
| Supplemental File 25 | Pathway analyses of secreted proteome | This file provides results of pathway analyses associated with the secreted proteome. The pathways listed are a combination of REACTOME pathways and our custom pathways and GOBP (Gene Ontology, Biological Processes). |
